# Supplementary material for: Social Isolation and Social Support Influence Health Service Utilisation and Survival after a Cardiovascular Disease Event: A Systematic Review
Source: Int J Environ Res Public Health. 2023 Mar 9;20(6):4853. doi: 10.3390/ijerph20064853 (PMC10049557; doi:10.3390/ijerph20064853)
Supplement: Supplementary file 1 [file ijerph-20-04853-s001.zip › ijerph-2206305-supplementary.pdf]

Supplementary S1: Search strategies

S1a. Embase from the earliest record to 21st June 2020

☐	1	social isolation/	24245	Advanced	Display Results | More ▾	
☐	2	social segregation/	184	Advanced	Display Results | More ▾	
☐	3	social distance/	2940	Advanced	Display Results | More ▾	
☐	4	loneliness/	8325	Advanced	Display Results | More ▾	
☐	5	psychosocial deprivation.mp.	147	Advanced	Display Results | More ▾	
☐	6	social participation/	6133	Advanced	Display Results | More ▾	
☐	7	community participation/	2284	Advanced	Display Results | More ▾	
☐	8	community integration/	1032	Advanced	Display Results | More ▾	
☐	9	social* isolat*.mp.	27891	Advanced	Display Results | More ▾	
☐	10	social* interact*.mp.	67044	Advanced	Display Results | More ▾	
☐	11	social* support*.mp.	102137	Advanced	Display Results | More ▾	
☐	12	social* disconnect*.mp.	166	Advanced	Display Results | More ▾	
☐	13	social* separat*.mp.	258	Advanced	Display Results | More ▾	
☐	14	social* segregat*.mp.	250	Advanced	Display Results | More ▾	
☐	15	social* exclu*.mp.	3245	Advanced	Display Results | More ▾	
☐	16	social* marginalis*.mp.	114	Advanced	Display Results | More ▾	
☐	17	social* alienat*.mp.	250	Advanced	Display Results | More ▾	
☐	18	social* communicat*.mp.	4562	Advanced	Display Results | More ▾	

☐	19	social* divid*.mp.	28	Advanced	Display Results | More ▾	🗨
☐	20	social* seclu*.mp.	13	Advanced	Display Results | More ▾	🗨
☐	21	social* detach*.mp.	46	Advanced	Display Results | More ▾	🗨
☐	22	support system.mp.	32422	Advanced	Display Results | More ▾	🗨
☐	23	support network.mp.	1924	Advanced	Display Results | More ▾	🗨
☐	24	solitude.mp.	624	Advanced	Display Results | More ▾	🗨
☐	25	social* cohesi*.mp.	1535	Advanced	Display Results | More ▾	🗨
☐	26	social* tie*.mp.	1020	Advanced	Display Results | More ▾	🗨
☐	27	social* embedd*.mp.	213	Advanced	Display Results | More ▾	🗨
☐	28	social* integrat*.mp.	3784	Advanced	Display Results | More ▾	🗨
☐	29	exp Australia/	171731	Advanced	Display Results | More ▾	🗨
☐	30	exp New Zealand/	60011	Advanced	Display Results | More ▾	🗨
☐	31	Australia*.mp.	272765	Advanced	Display Results | More ▾	🗨
☐	32	New Zealand*.mp.	114136	Advanced	Display Results | More ▾	🗨
☐	33	Maori*.mp.	4602	Advanced	Display Results | More ▾	🗨
☐	34	Aboriginal*.mp.	11502	Advanced	Display Results | More ▾	🗨
☐	35	Torres Strait Islander*.mp.	2181	Advanced	Display Results | More ▾	🗨
☐	36	Indigenous*.mp.	43496	Advanced	Display Results | More ▾	🗨

37	ATSI.mp.	124	Advanced	Display Results | More	
38	social support/	90575	Advanced	Display Results | More	
39	aotearoa*.mp.	498	Advanced	Display Results | More	
40	cardiovascular disease/	277325	Advanced	Display Results | More	
41	cardiovascular disease*.tw.	245963	Advanced	Display Results | More	
42	cardiovascular.mp.	1053499	Advanced	Display Results | More	
43	CVD.mp.	56675	Advanced	Display Results | More	
44	heart disease/	127875	Advanced	Display Results | More	
45	coronary disease.mp.	28643	Advanced	Display Results | More	
46	coronary heart disease*.mp.	72891	Advanced	Display Results | More	
47	myocardial infarction*.mp.	302976	Advanced	Display Results | More	
48	heart attack*.mp.	8271	Advanced	Display Results | More	
49	stroke.mp.	449558	Advanced	Display Results | More	
50	heart failure/	249444	Advanced	Display Results | More	
51	coronary artery disease/	206945	Advanced	Display Results | More	
52	myocardial ischemia.mp.	41760	Advanced	Display Results | More	
53	myocardial ischaemia.mp.	7433	Advanced	Display Results | More	
54	hypertensive heart disease*.mp.	2825	Advanced	Display Results | More	

☐	55	angina.mp.	120049	Advanced	Display Results | More	
☐	56	angina pectoris/	68267	Advanced	Display Results | More	
☐	57	vascular disease/	69541	Advanced	Display Results | More	
☐	58	((Cardiovascular or cardio-vascular) adj3 (disease* or disorder* or condition*)).tw.	276084	Advanced	Display Results | More	
☐	59	((Coronary or heart or cardiac or myocardi*) adj3 (disease* or disorder* or condition* or infarct*)).tw.	700503	Advanced	Display Results | More	
☐	60	brain ischemia/	142426	Advanced	Display Results | More	
☐	61	brain ischaemia.mp.	723	Advanced	Display Results | More	
☐	62	cerebrovascular accident/	208472	Advanced	Display Results | More	
☐	63	social* distan*.mp.	4183	Advanced	Display Results | More	
☐	64	1 or 2 or 3 or 4 or 5 or 6 or 7 or 8 or 9 or 10 or 11 or 12 or 13 or 14 or 15 or 16 or 17 or 18 or 19 or 20 or 21 or 22 or 23 or 24 or 25 or 26 or 27 or 28 or 38 or 63	248241	Advanced	Display Results | More	
☐	65	29 or 30 or 31 or 32 or 33 or 34 or 35 or 36 or 37 or 39	390014	Advanced	Display Results | More	
☐	66	40 or 41 or 42 or 43 or 44 or 45 or 46 or 47 or 48 or 49 or 50 or 51 or 52 or 53 or 54 or 55 or 56 or 57 or 58 or 59 or 60 or 61 or 62	2330475	Advanced	Display Results | More	
☐	67	64 and 65 and 66	395	Advanced	Display Results | More	

S1b. MEDLINE from the earliest record to 21st June 2020

1	Social Isolation/	13200	Advanced	Display Results | More	
2	Social Segregation/	131	Advanced	Display Results | More	
3	Social Distance/	2878	Advanced	Display Results | More	
4	Social Alienation/	1388	Advanced	Display Results | More	
5	Loneliness/	3661	Advanced	Display Results | More	
6	Psychosocial Deprivation/	1996	Advanced	Display Results | More	
7	Social Participation/ or Community Participation/	19419	Advanced	Display Results | More	
8	Community Integration/	344	Advanced	Display Results | More	
9	social* isolat*.mp.	16835	Advanced	Display Results | More	
10	social* interact*.mp.	16446	Advanced	Display Results | More	
11	social* support*.mp.	82978	Advanced	Display Results | More	
12	social* disconnect*.mp.	93	Advanced	Display Results | More	
13	social* separat*.mp.	182	Advanced	Display Results | More	
14	social* segregat*.mp.	189	Advanced	Display Results | More	
15	social* exclu*.mp.	1572	Advanced	Display Results | More	
16	social* marginalis*.mp.	64	Advanced	Display Results | More	
17	social* alienat*.mp.	1472	Advanced	Display Results | More	
18	social* communicat*.mp.	2601	Advanced	Display Results | More	

19	social* divid*.mp.	22	Advanced	Display Results | More	
20	social* seclu*.mp.	6	Advanced	Display Results | More	
21	social* detach*.mp.	32	Advanced	Display Results | More	
22	support system.mp.	6617	Advanced	Display Results | More	
23	support network.mp.	1143	Advanced	Display Results | More	
24	solitude.mp.	344	Advanced	Display Results | More	
25	social* cohesi*.mp.	1097	Advanced	Display Results | More	
26	social* tie*.mp.	736	Advanced	Display Results | More	
27	social* embedd*.mp.	157	Advanced	Display Results | More	
28	social* integrat*.mp.	2354	Advanced	Display Results | More	
29	exp Australia/	143439	Advanced	Display Results | More	
30	exp New Zealand/	39132	Advanced	Display Results | More	
31	Australia*.mp.	163573	Advanced	Display Results | More	
32	New Zealand*.mp.	65043	Advanced	Display Results | More	
33	Maori*.mp.	3103	Advanced	Display Results | More	
34	Aboriginal*.mp.	7886	Advanced	Display Results | More	
35	Torres Strait Islander*.mp.	1310	Advanced	Display Results | More	
36	Indigenous*.mp.	28570	Advanced	Display Results | More	

☐	37	ATSI.mp.	54	Advanced	Display Results | More ▾	🗨
☐	38	Social Support/	70251	Advanced	Display Results | More ▾	🗨
☐	39	aotearoa*.mp.	322	Advanced	Display Results | More ▾	🗨
☐	40	Cardiovascular Diseases/	147352	Advanced	Display Results | More ▾	🗨
☐	41	cardiovascular disease*.tw.	143104	Advanced	Display Results | More ▾	🗨
☐	42	cardiovascular.mp.	509330	Advanced	Display Results | More ▾	🗨
☐	43	CVD.mp.	27638	Advanced	Display Results | More ▾	🗨
☐	44	Heart Diseases/	69218	Advanced	Display Results | More ▾	🗨
☐	45	Coronary Disease/	130707	Advanced	Display Results | More ▾	🗨
☐	46	coronary heart disease*.mp.	46536	Advanced	Display Results | More ▾	🗨
☐	47	Myocardial Infarction/	165144	Advanced	Display Results | More ▾	🗨
☐	48	heart attack*.mp.	4859	Advanced	Display Results | More ▾	🗨
☐	49	Stroke/	100731	Advanced	Display Results | More ▾	🗨
☐	50	Heart Failure/	117748	Advanced	Display Results | More ▾	🗨
☐	51	Coronary Artery Disease/	61642	Advanced	Display Results | More ▾	🗨
☐	52	Myocardial Ischemia/	38554	Advanced	Display Results | More ▾	🗨
☐	53	myocardial ischaemia.mp.	4848	Advanced	Display Results | More ▾	🗨
☐	54	hypertensive heart disease*.mp.	1480	Advanced	Display Results | More ▾	🗨

55	Angina Pectoris/	32432	Advanced	Display Results | More	
56	angina.mp.	66079	Advanced	Display Results | More	
57	Vascular Diseases/	36395	Advanced	Display Results | More	
58	((cardiovascular or cardio-vascular) adj3 (disease* or disorder* or condition*)).tw.	159951	Advanced	Display Results | More	
59	((Coronary or heart or cardiac or myocardi*) adj3 (disease* or disorder* or condition* or infarct*)).tw.	416503	Advanced	Display Results | More	
60	Brain Ischemia/	51871	Advanced	Display Results | More	
61	brain ischaemia.mp.	460	Advanced	Display Results | More	
62	cerebrovascular accident*.tw.	6018	Advanced	Display Results | More	
63	social* distan*.mp.	3702	Advanced	Display Results | More	
64	1 or 2 or 3 or 4 or 5 or 6 or 7 or 8 or 9 or 10 or 11 or 12 or 13 or 14 or 15 or 16 or 17 or 18 or 19 or 20 or 21 or 22 or 23 or 24 or 25 or 26 or 27 or 28 or 38 or 63	152554	Advanced	Display Results | More	
65	29 or 30 or 31 or 32 or 33 or 34 or 35 or 36 or 37 or 39	258302	Advanced	Display Results | More	
66	40 or 41 or 42 or 43 or 44 or 45 or 46 or 47 or 48 or 49 or 50 or 51 or 52 or 53 or 54 or 55 or 56 or 57 or 58 or 59 or 60 or 61 or 62	1253900	Advanced	Display Results | More	
67	64 and 65 and 66	135	Advanced	Display Results | More	

S1c. Scopus from the earliest record to 21st June 2020

431 document results

((TITLE-ABS-KEY ("social* isolat*" OR "social* interact*" OR "social* support*") OR TITLE-ABS-KEY ("social* disconnect*" OR "social* separat*" OR "social* segregat*") OR TITLE-ABS-KEY ("social* exclu*" OR "social* distan*" OR "social* marginalis*") OR TITLE-ABS-KEY ("social* alienat*" OR "social* communicat*" OR "social* divid*") OR TITLE-ABS-KEY ("social* seclu*" OR "social* detach*" OR "support system") OR TITLE-ABS-KEY ("support network" OR "loneliness" OR "solitude") OR TITLE-ABS-KEY ("psychosocial deprivation" OR "social participation" OR "community participation") OR TITLE-ABS-KEY ("social* cohesi*" OR "social* tie*" OR "social* embedd*") OR TITLE-ABS-KEY ("community integration" OR "social* integrat*"))) AND ((TITLE-ABS-KEY (australia* OR "new zealand*") OR TITLE-ABS-KEY (maori* OR aboriginal* OR indigenous*) OR TITLE-ABS-KEY ("torres strait islander" OR atsi OR aotearoa*))) AND ((TITLE-ABS-KEY ("cardiovascular disease*" OR "cardiovascular" OR cvd) OR TITLE-ABS-KEY ("heart disease*" OR "coronary disease*" OR "coronary heart disease*") OR TITLE-ABS-KEY ("myocardial infarction*" OR "heart attack*" OR stroke OR "heart failure") OR TITLE-ABS-KEY ("coronary artery disease" OR "myocardial ischemia" OR "myocardial ischaemia" OR "hypertensive heart disease*") OR TITLE-ABS-KEY ("angina" OR "angina pectoris" OR "vascular disease*") OR TITLE-ABS-KEY ("(Cardiovascular or cardio-vascular) adj3 (disease* or disorder* or condition*)") OR TITLE-ABS-KEY ("(Coronary or heart or cardiac or myocardi*) adj3 (disease* or disorder* or condition* or infarct*)") OR TITLE-ABS-KEY ("brain ischemia" OR "brain ischaemia" OR "cerebrovascular accident*")))

S1d. Web of Science from the earliest record to 21st June 2020

Set	Results	Save History / Create Alert / Open Saved History	Edit Sets	Combine Sets (AND / OR, Combine)	Delete Sets (Select All, Delete)
# 7	168	#5 AND #3 AND #2<br>*Indexes=SCI-EXPANDED, SSCI, A&HCI, CPCI-S, CPCI-SSH, BKCI-S, BKCI-SSH, ESCI, CCR-EXPANDED, IC Timespan=All years*	Edit		
# 6	82	#5 AND #4<br>*Indexes=SCI-EXPANDED, SSCI, A&HCI, CPCI-S, CPCI-SSH, BKCI-S, BKCI-SSH, ESCI, CCR-EXPANDED, IC Timespan=All years*	Edit		
# 5	1,567,406	**TOPIC:** ("cardiovascular disease*" OR "cardiovascular" OR CVD) *OR* **TOPIC:** ("heart disease*" OR "coronary disease*" OR "coronary heart disease*") *OR* **TOPIC:** ("myocardial infarction*" OR "heart attack*" OR stroke OR "heart failure") *OR* **TOPIC:** ("coronary artery disease" OR "myocardial ischemia" OR "myocardial ischaemia" OR "hypertensive heart disease*") *OR* **TOPIC:** ("angina" OR "angina pectoris" OR "vascular disease*") *OR* **TOPIC:** ("(Cardiovascular or cardio-vascular) adj3 (disease* or disorder* or condition*) ") *OR* **TOPIC:** ("(Coronary or heart or cardiac or myocardi*) adj3 (disease* or disorder* or condition* or infarct*) ") *OR* **TOPIC:** ("brain ischemia" OR "brain ischaemia" OR "cerebrovascular accident*")<br>*Indexes=SCI-EXPANDED, SSCI, A&HCI, CPCI-S, CPCI-SSH, BKCI-S, BKCI-SSH, ESCI, CCR-EXPANDED, IC Timespan=All years*	Edit		
# 4	1,498	#3 AND #2 AND #1<br>*Indexes=SCI-EXPANDED, SSCI, A&HCI, CPCI-S, CPCI-SSH, BKCI-S, BKCI-SSH, ESCI, CCR-EXPANDED, IC Timespan=All years*	Edit		
# 3	602,203	TS=(australia* OR "new zealand*") OR TS=(maori* OR aboriginal* OR indigenous*) OR TS=("torres strait islander" OR atsi OR aotearoa*)<br>*Indexes=SCI-EXPANDED, SSCI, A&HCI, CPCI-S, CPCI-SSH, BKCI-S, BKCI-SSH, ESCI, CCR-EXPANDED, IC Timespan=All years*	Edit		
# 2	230,539	**TOPIC:** ("social* isolat*" OR "social* interact*" OR "social* support*") *OR* **TOPIC:** ("social* disconnect*" OR "social* separat*" OR "social* segregat*") *OR* **TOPIC:** ("social* exclu*" OR "social* distan*" OR "social* marginalis*") *OR* **TOPIC:** ("social* alienat*" OR "social* communicat*" OR "social* divid*") *OR* **TOPIC:** ("social* seclu*" OR "social* detach*" OR "support system") *OR* **TOPIC:** ("support network" OR "loneliness" OR "solitude") *OR* **TOPIC:** ("psychosocial deprivation" OR "social participation" OR "community participation") *OR* **TOPIC:** ("social* cohesi*" OR "social* tie*" OR "social* embedd*") *OR* **TOPIC:** ("community integration" OR "social* integrat*")<br>*Indexes=SCI-EXPANDED, SSCI, A&HCI, CPCI-S, CPCI-SSH, BKCI-S, BKCI-SSH, ESCI, CCR-EXPANDED, IC Timespan=All years*	Edit		
# 1	3,508,781	TS=("old* adult*" OR aged OR elderly OR "Aged 65") OR TS=("late adult*" OR geriatrics OR "geriatric community" OR "geriatric population") OR TS=("aged 80, and over" OR "frail elderly") OR TS=("old* citizen*1" OR "senior citizen*")<br>*Indexes=SCI-EXPANDED, SSCI, A&HCI, CPCI-S, CPCI-SSH, BKCI-S, BKCI-SSH, ESCI, CCR-EXPANDED, IC Timespan=All years*	Edit		
				AND / OR<br>Combine	Select All<br>Delete

Supplementary S2: Studies awaiting classification

Beath, A., et al. (2011). "Clinical predictors of outcome in acute ischaemic stroke patients treated with intravenous tissue plasminogen activator (tPA)." International Journal of Stroke 6: 4.

Long, P. W., et al. (2001). "Facilitating best practice: Transferring the lessons of the Clinical Support Systems Program." Journal of Quality in Clinical Practice 21(4): 157-159.

McDonnell, M. N. (2010). "Management of cognitive impairment following stroke: An audit of australian rehabilitation units." Cerebrovascular Diseases 29(SUPPL. 2): 69.

McLachlan, A., et al. (2010). "Nurse led quality improvement initiatives improving uptake of cardiac rehabilitation." Heart Lung and Circulation 19(SUPPL. 2): S245-S246.

Nilsson, M. and T. Linden (2009). "Multimodal sensory stimulation in stroke rehabilitation: The culture and brain health project." International Journal of Stroke 4(SUPPL. 1): 19.

Power, L. and M. Kennedy-Jones (2015). "The experience of early supported discharge for stroke survivors." International Journal of Stroke 10(SUPPL. 3): 75.

Riddell, T. (2011). "Inequities in Maori cardiovascular health require combined population and clinical health responses." Heart Lung and Circulation 20(SUPPL. 1): S4.

Sanders, L., et al. (2009). "An alternative outpatient based model of transient ischaemia attack management - The Monash TIA triaging treatment system." International Journal of Stroke 4(SUPPL. 1): 11.

Zecchin, R., et al. (2016). "Cardiac rehabilitation for patients with spontaneous coronary artery dissection." Heart Lung and Circulation 25(Supplement 2): S324.

Supplementary S3: Excluded Papers

Study	Title	DOI	Source	Exclusion Reason
Abbott 2010	Barriers and enhancers to dietary behaviour change for Aboriginal people attending a diabetes cooking course		Main search	02. Not CVD;
Abimbola 2019	The NASSS framework for ex post theorisation of technology-supported change in healthcare: Worked example of the TORPEDO programme	10.1186/s12916-019-1463-x	Main search	03. Not SH;
Adams 2009	Effects of area deprivation on health risks and outcomes: a multilevel, cross-sectional, Australian population study	10.1007/s00038-009-7113-x	Main search	03. Not SH;

Albarqouni 2019	External validation and comparison of four cardiovascular risk prediction models with data from the Australian Diabetes, Obesity and Lifestyle study	10.5694/mja2.12061	Main search	03. Not SH;
Alfonso 2012	Perception of worsening health predicts mortality in older men: The Health in Men Study (HIMS)	10.1016/j.archger.2012.04.005	Main search	04. CVD & SH mentioned but no outcome;
Al-Ganmi	Medication adherence and predictive factors in patients with cardiovascular disease: A cross-sectional study	10.1111/nhs.12681	Main search	07. Outcome not hospital utilisation
Allen 2013	Quality of life impact of cardiovascular and affective conditions among older residents from urban and rural communities	10.1186/1477-7525-11-140	Main search	04. CVD & SH

				mentioned but no outcome;
Allisey 2016	An application of an extended effort-reward imbalance model to police absenteeism behaviour	10.1108/pr-06-2014-0125	Main search	02. Not CVD;
Almeida 2005	Depression and smoking amongst older general practice patients	10.1016/j.jad.2005.02.014	Main search	02. Not CVD;
Almeida 2011	A practical approach to assess depression risk and to guide risk reduction strategies in later life	10.1017/S1041610210001870	Main search	04. CVD & SH mentioned but no outcome;

Almeida 2011	Complaints of difficulty to fall asleep increase the risk of depression in later life: the health in men study	10.1016/j.jad.2011.05.045	Main search	04. CVD & SH mentioned but no outcome;
Almeida 2012	Cardiovascular disease, depression and mortality: the Health In Men Study	10.1097/JGP.0b013e318211c1ed	Main search	04. CVD & SH mentioned but no outcome;
Almeida 2013	Cardiovascular diseases do not influence the mental health outcome of older men with depression over 6 years	10.1016/j.jad.2012.06.043	Main search	04. CVD & SH mentioned

			but no outcome;
Andersson 1995	A population-based assessment of the impact and burden of caregiving for long-term stroke survivors	Main search	04. CVD & SH mentioned but no outcome;
Andersson 1996	Validation of the Short Form 36 (SF-36) health survey questionnaire among stroke patients	Main search	04. CVD & SH mentioned but no outcome;

Anderson 2004	The postmodern heart: War veterans' experiences of invasive cardiac technology	10.1111/j.1365-2648.2004.02985.x	Main search	04. CVD & SH mentioned but no outcome;
Anderson 2006	The effects of a multimodal intervention trial to promote lifestyle factors associated with the prevention of cardiovascular disease in menopausal and postmenopausal Australian women	10.1080/07399330500506543	Main search	04. CVD & SH mentioned but no outcome;
Andrew 2013	Differences in long-term unmet needs between younger and older stroke survivors	10.1159/000353129	Main search	07. Outcome not hospital utilisation

Andrew 2013	Long-term unmet needs of community dwelling stroke survivors and carers in Australia	10.1159/000353129	Main search	07. Outcome not hospital utilisation
Andrew 2013	Understanding the factors associated with unmet needs in Australian stroke survivors	10.1111/ijs.12142	Main search	07. Outcome not hospital utilisation
Andrew 2013	The impact of stroke survivor disability on the long-term needs of carers	10.1111/ijs.12172	Main search	04. CVD & SH mentioned but no outcome;

Andrew 2014	Understanding long-term unmet needs in Australian survivors of stroke	10.1111/ijs.12325	Main search	07. Outcome not hospital utilisation
Aoun 2017	Bereavement support for family caregivers: The gap between guidelines and practice in palliative care	10.1371/journal.pone.0184750	Main search	02. Not CVD
Armstrong 2012	Living with aphasia: three Indigenous Australian stories	10.3109/17549507.2011.663790	Main search	02. Not CVD;
Arndt 2009	"Others had similar problems and you were not alone": Evaluation of an open-group mutual aid model in cardiac rehabilitation	10.1097/JCN.0b013e3181a1c236	Main search	04. CVD & SH mentioned but no outcome;

Arroll 2009	Managing Cardiovascular Risk in the Future	10.1002/9781444303353.ch12	Main search	03. Not SH;
Aslani 2011	A community pharmacist delivered adherence support service for dyslipidaemia	10.1093/eurpub/ckq118	Main search	02. Not CVD;
Aspin 2012	Strategic approaches to enhanced health service delivery for Aboriginal and Torres Strait Islander people with chronic illness: A qualitative study	10.1186/1472-6963-12-143	Main search	02. Not CVD;
Astley 2011	Health resource variability in the achievement of optimal performance and clinical outcome	10.1161/CIRCOUTCOMES.110.960229	Main search	03. Not SH;
Attard 2012	The comparative effects of multi-modality aphasia therapy and constraint-induced aphasia therapy-plus treatments for severe chronic Broca's aphasia: A pilot study		Main search	02. Not CVD

Azzopardi 2009	Health-Related Quality of Life 2 Years After Coronary Artery Bypass Graft Surgery	10.1097/JCN.0b013e31819b2125	Main search	04. CVD & SH mentioned but no outcome;
Badcock 2018	Loneliness in psychotic illness and its association with cardiometabolic disorders	10.1016/j.schres.2018.09.021	Main search	02. Not CVD;
Badcock 2019	Loneliness in psychotic illness and its association with cardiometabolic disorders	10.1016/j.schres.2018.09.021	Main search	02. Not CVD;
Bagot 2017	Integrating acute stroke telemedicine consultations into specialists' usual practice: a qualitative analysis comparing the experience of Australia and the United Kingdom	10.1186/s12913-017-2694-1	Main search	04. CVD & SH mentioned

				but no outcome;
Bailie 2017	Improving preventive health care in Aboriginal and Torres Strait Islander primary care settings	10.1186/s12992-017-0267-z	Main search	02. Not CVD;
Baker 2019	Risk factors for acute rheumatic fever: Literature review and protocol for a case-control study in New Zealand	10.3390/ijerph16224515	Main search	06.Review;
Bannink 2006	Web-based assessment of cardiovascular disease risk in routine primary care practice in New Zealand: The first 18,000 patients (PREDICT CVD-1)		Main search	03. Not SH;
Barker 2005	Upper limb recovery after stroke: the stroke survivors' perspective		Main search	07. Outcome not hospital utilisation
Bean 2007	Ethnic differences in illness perceptions, self-efficacy and diabetes self-care	10.1080/14768320600976240	Main search	02. Not CVD;

Beard 2009	Influence of socioeconomic and cultural factors on rural health	10.1111/j.1440-1584.2008.01030.x	Main search	02. Not CVD;
Beckley 2007	The influence of the quality and quantity of social support in the promotion of community participation following stroke	10.1111/j.1440-1630.2007.00643.x	Main search	01. Not AUS/NZ;
Beesley 2011	Art after stroke: the qualitative experience of community dwelling stroke survivors in a group art programme	10.3109/09638288.2011.571333	Main search	05. Intervention;
Bertorelli 2015	Bertorelli, D. (2015). "From the mind to the heart: Cardio-psychiatry or psycho-cardiology?" Australian and New Zealand Journal of Psychiatry 49: 75.		Main search	03. Not SH;
Billah 2014	AusSCORE II in predicting 30-day mortality after isolated coronary artery bypass grafting in Australia and New Zealand	10.1016/j.jtcvs.2014.02.027	Main search	03. Not SH;

Blacker 2010	Evaluation of the effectiveness of an acceptance and commitment therapy (ACT) groupprogram to improve coping and quality of life for individuals post stroke	10.1111/j.1747-4949.2010.00458-4.x	Main search	05. Intervention;
Blacker 2019	Indigenous stroke care: differences, challenges and a need for change	10.1111/imj.14399	Main search	04. CVD & SH mentioned but no outcome;
Blomqvist 2018	Enabling healthy living: Experiences of people with severe mental illness in psychiatric outpatient services	10.1111/inm.12313	Main search	02. Not CVD;

Bogomolova 2018	Dietary intervention for people with mental illness in South Australia	10.1093/heapro/daw055	Main search	02. Not CVD;
Bonner 2019	Implementing cardiovascular disease prevention guidelines to translate evidence-based medicine and shared decision making into general practice: Theory-based intervention development, qualitative piloting and quantitative feasibility	10.1186/s13012-019-0927-x	Main search	03. Not SH;
Booth 1997	Physical activity preferences, preferred sources of assistance, and perceived barriers to increased activity among physically inactive Australians		Main search	02. Not CVD;
Boult 2011	Developing tools to predict outcomes following cardiovascular surgery	10.1111/j.1445-2197.2010.05644.x	Main search	03. Not SH;

Bradshaw 2015	Validation study of GRACE risk scores in indigenous and non-indigenous patients hospitalized with acute coronary syndrome	10.1186/s12872-015-0138-6	Main search	03. Not SH;
Bramley 2004	A call to action on Maori cardiovascular health		Main search	03. Not SH;
Brauer 2018	Improving physical activity after stroke via treadmill training and self management (IMPACT): A protocol for a randomised controlled trial	10.1186/s12883-018-1015-6	Main search	03. Not SH;
Brazionis 2017	An evaluation of the telehealth facilitation of diabetes and cardiovascular care in remote Australian Indigenous communities: - protocol for the telehealth eye and associated medical services network [TEAMSnet] project, a pre-post study design	10.1186/s12913-016-1967-4	Main search	02. Not CVD

Broad 2007	Zero end-digit preference in recorded blood pressure and its impact on classification of patients for pharmacologic management in primary care - PREDICT-CVD-6	10.3399/096016407782317964	Main search	03. Not SH;
Brodaty 2007	Rates of depression at 3 and 15 months poststroke and their relationship with cognitive decline: The Sydney stroke study	10.1097/JGP.0b013e3180590bca	Main search	07. Outcome not hospital utilisation
Brown 2006	Uncovering the determinants of cardiovascular disease among Indigenous people	10.1080/13557850500485485	Main search	04. CVD & SH mentioned but no outcome;
Brown 2013	Making a good time": The role of friendship in living successfully with aphasia	10.3109/17549507.2012.692814	Main search	02. Not CVD;

Brundisini 2013	Chronic disease patients' experiences with accessing health care in rural and remote areas: a systematic review and qualitative meta-synthesis		Main search	01. Not AUS/NZ;
Bulman 2011	Mibbinbah and spirit healing: Fostering safe, friendly spaces for indigenous males in Australia	10.3149/jmh.1001.6	Main search	02. Not CVD;
Bunani 2012	The association between social support and psychosocial factors upon mortality and quality of life	10.1111/j.1440-1797.2012.01633.x	Main search	01. Not AUS/NZ;
Bunker 2003	"Stress" and coronary heart disease: psychosocial risk factors		Main search	06.Review;
Burgess 2015	Strengthening Cardiovascular Disease Prevention in Remote Indigenous Communities in Australia's Northern Territory	10.1016/j.hlc.2014.11.008	Main search	05. Intervention;

Butland 2019	Health behaviours of rural Australians following percutaneous coronary intervention: a systematic scoping review	10.22605/RRH4854	Main search	06.Review;
Byard 2002	Incapacitation or death of a socially isolated parent or carer could result in the death of dependent children	10.1046/j.1440-1754.2002.00025.x	Main search	02. Not CVD;
Byles 2014	PSYCHOLOGICAL DISTRESS AND COMORBID PHYSICAL CONDITIONS: DISEASE OR DISABILITY?	10.1002/da.22162	Main search	04. CVD & SH mentioned but no outcome;
Byles 2015	Long-term survival of older australian women with a history of stroke	10.1016/j.jstrokecerebrovasdis.2014.07.040	Main search	04. CVD & SH mentioned

				but no outcome;
Cadden 2007	Educating nurses about cardiac monitoring in a stroke unit		Main search	03. Not SH;
Cadilhac 2017	Improving discharge care: The potential of a new organisational intervention to improve discharge after hospitalisation for acute stroke, a controlled before-after pilot study	10.1136/bmjopen-2017-016010	Main search	05. Intervention;
Cameron 2008			References	07. Outcome not hospital utilisation
Cameron 2010	Does cognitive impairment predict poor self-care in patients with heart failure?	10.1093/eurjhf/hfq042	Main search	04. CVD & SH mentioned

				but no outcome;
Cameron 2016	Psychosocial adjustment of patients living with an internal cardioverter defibrillator	10.1093/eurheartj/ehw433	Main search	07. Outcome not hospital utilisation
Cameron 2016	Carers' views on patient self-care in chronic heart failure	10.1111/jocn.13124	Main search	07. Outcome not hospital utilisation
Campbell 1994	Disease, impairment, disability and social handicap: A community based study of people aged 70 years and Over	10.3109/09638289409166015	Main search	04. CVD & SH mentioned but no outcome;

Canuto 2011	Study protocol: a pragmatic randomised controlled trial of a 12-week physical activity and nutritional education program for overweight Aboriginal and Torres Strait Islander women	10.1186/1471-2458-11-655	Main search	02. Not CVD;
Cape 1994	The influence of clinical problems, age and social support on outcomes for elderly persons referred to regional aged care assessment teams		Main search	02. Not CVD;
Caperchione 2011	Physical activity behaviours of Culturally and Linguistically Diverse (CALD) women living in Australia: a qualitative study of socio-cultural influences	10.1186/1471-2458-11-26	Main search	02. Not CVD;
Caspi 2006	Socially isolated children 20 years later - Risk of cardiovascular disease	10.1001/archpedi.160.8.805	Main search	02. Not CVD;
Cassel 1974	Hypertension and cardiovascular disease in migrants: a potential source of clues?		Main search	04. CVD & SH mentioned

				but no outcome;
Chan 1285	Ethnic and socioeconomic disparities in the prevalence of cardiovascular disease in New Zealand		Main search	03. Not SH;
Chan 2008	Ethnic and socioeconomic disparities in the prevalence of cardiovascular disease in New Zealand	10.1136/bmj.39455.596181.25	Main search	04. CVD & SH mentioned but no outcome;
Cheok 2003			References	07. Outcome not hospital utilisation

Cheok 2003	Identification, course, and treatment of depression after admission for a cardiac condition: Rationale and patient characteristics for the Identifying Depression As a Comorbid Condition (IDACC) project	10.1016/s0002-8703(03)00481-2	Main search	07. Outcome not hospital utilisation
Clark 1998	The effects of depression and abnormal illness behaviour on outcome following rehabilitation from stroke	10.1191/026921598669567216	Main search	03. Not SH;
Clark 1999	Psychological correlates of outcome following rehabilitation from stroke	10.1191/026921599673399613	Main search	03. Not SH;
Clark 1999	Changes in family functioning for stroke rehabilitation patients and their families	10.1097/00004356-199909000-00003	Main search	04. CVD & SH mentioned but no outcome;

Clark 2003	A randomized controlled trial of an education and counselling intervention for families after stroke		Main search	05. Intervention;
Clark 2010	Home based cardiac rehabilitation	10.1136/bmj.b5510	Main search	06.Review;
Clark 2015	Development and feasibility testing of an education program to improve knowledge and self-care among Aboriginal and Torres Strait Islander patients with heart failure		Main search	03. Not SH;
Cleland 2010	Individual, social and environmental correlates of physical activity among women living in socioeconomically disadvantaged neighbourhoods	10.1016/j.socscimed.2010.02.028	Main search	02. Not CVD;
Courtney 2009	Fewer Emergency Readmissions and Better Quality of Life for Older Adults at Risk of Hospital Readmission: A Randomized Controlled Trial to Determine the Effectiveness of a 24-Week Exercise and Telephone Follow-Up Program	10.1111/j.1532-5415.2009.02138.x	Main search	05. Intervention;

Cruice 2010	Health-related quality of life in people with aphasia: Implications for fluency disorders quality of life research	10.1016/j.jfludis.2010.05.008	Main search	02. Not CVD;
Daly 2000	Health status, perceptions of coping, and social support immediately after discharge of survivors of acute myocardial infarction		Main search	04. CVD & SH mentioned but no outcome;
Danese 2009	Adverse childhood experiences and adult risk factors for age-related disease: depression, inflammation, and clustering of metabolic risk markers	10.1001/archpediatrics.2009.214	Main search	02. Not CVD;
Daniel 2011	Environmental risk conditions and pathways to cardiometabolic diseases in indigenous populations	10.1146/annurev.publhealth.012809.103557	Main search	04. CVD & SH mentioned

			but no outcome;
Davidson 2003	Identifying the communication activities of older people with aphasia: Evidence from naturalistic observation	Main search	02. Not CVD;
Davidson 2004	Integrated, collaborative palliative care in heart failure: the St. George Heart Failure Service experience 1999-2002	Main search	04. CVD & SH mentioned but no outcome;
Davidson 2005	Activities of home-based heart failure nurse specialists: a modified narrative analysis	Main search	04. CVD & SH mentioned

				but no outcome;
Davidson 2006	Social communication in older age: Lessons from people with aphasia	10.1310/0GGQ-CJDX-N2BR-W7W4	Main search	02. Not CVD;
Davidson 2008	Social participation for older people with aphasia: The impact of communication disability on friendships	10.1310/tsr1504-325	Main search	02. Not CVD;
Dean 2009	Exercise intervention to prevent falls and enhance mobility in community dwellers after stroke: a protocol for a randomised controlled trial	10.1186/1471-2377-9-38	Main search	05. Intervention;

Dean 2014	Treadmill training provides greater benefit to the subgroup of community-dwelling people after stroke who walk faster than 0.4 m/s: a randomised trial	10.1016/j.jphys.2014.03.004	Main search	05. Intervention;
Dengler 2011	The heart beads program	10.1111/j.1744-6155.2010.00273.x	Main search	04. CVD & SH mentioned but no outcome;
Denham 2019	"This is our life now. our new normal": A qualitative study of the unmet needs of carers of stroke survivors	10.1177/1747493019858233	Main search	04. CVD & SH mentioned but no outcome;

DiBenedetto 2007			References	07. Outcome not hospital utilisation
DiBenedetto 2010	A biopsychosocial model for depressive symptoms following acute coronary syndromes	10.1080/08870440903019535	Main search	04. CVD & SH mentioned but no outcome;
Dollard 2004	Broadening the reach of cardiac rehabilitation to rural and remote Australia		Main search	06.Review;
D'Onise 2012	Does an early childhood intervention affect cardiometabolic risk in adulthood? Evidence from a longitudinal study of preschool attendance in South Australia	10.1016/j.puhe.2012.04.012	Main search	02. Not CVD;

Dracup 2006	A nursing intervention to reduce prehospital delay in acute coronary syndrome: a randomized clinical trial		Main search	05. Intervention;
Draper 2007	Stress in caregivers of aphasic stroke patients: A randomized controlled trial	10.1177/0269215506071251	Main search	05. Intervention;
DuPlessis 2018	Traversing the liminal: What can Fontan adults' transition experiences and perspectives teach us about optimizing healthcare?	10.1515/ijamh-2018-0020	Main search	04. CVD & SH mentioned but no outcome;
Dyall 2008	Stroke: A picture of health disparities in New Zealand		Main search	04. CVD & SH mentioned

				but no outcome;
Essue 2010	Informal care and the self-management partnership: Implications for Australian health policy and practice	10.1071/AH09795 10.1207/S15324796ABM2601-01	Main search	04. CVD & SH mentioned but no outcome;
Feibel 1982			References	01. Not AUS/NZ;
Ferry 2004	Towards a safer culture: Clinical pathways in acute coronary syndromes and stroke		Main search	03. Not SH;

Field 2002	A case study in strategic change: developing a strategic research program to address cardiovascular disease and related disorders in aboriginal and Torres Strait Islander peoples and rural and remote settings		Main search	03. Not SH;
Filip 1988	Filip J, Rubenfire M, Mosca L. Effect of gender and age on barriers to participation on cardiac rehabilitation following an acute myocardial infarction. CVD Prev 1998; 1:299 – 305.		References	01. Not AUS/NZ;
Finch 2017	Undetected and underserved: The untold story of patients who had a minor stroke: Equity of access is particularly concerning for minor stroke	10.5694/mja16.01009	Main search	06.Review;
Fini 2014	How physically active are people following stroke?	10.1111/ijs.12297	Main search	06.Review;

Friedland 1987	Friedland,J., & McColl, M. A. (1987). Social support and psychosocial dysfunction after stroke: Buffering effects in a community Archives of Physical Medicine and Rehabilitation. 68,475-480.		References	01. Not AUS/NZ;
Fullagar 2003	Governing women's active leisure: The gendered effects of calculative rationalities within Australian health policy	10.1080/0958159031000100206	Main search	02. Not CVD;
Gallagher 2003	Effects of a telephone counseling intervention on psychosocial adjustment in women following a cardiac event		Main search	05. Intervention;
Gallagher 2012	Weight management issues and strategies for people with high cardiovascular risk undertaking an Australian weight loss program: a focus group study	10.1111/j.1442-2018.2011.00651.x	Main search	02. Not CVD;

Gallagher 2016	Quality of life, social support and cognitive impairment in heart failure patients without diagnosed dementia	10.1111/ijn.12402	Main search	07. Outcome not hospital utilisation
Garcia 2019	The roles of dispositional coping style and social support in helping people with respiratory disease cope with a breathlessness crisis	10.1111/jan.14039	Main search	02. Not CVD;
Garofalo 2012	Pre-hospital delay in acute coronary syndromes: PREDICT CVD-18		Main search	03. Not SH;
Gaskin 2015	Parents experiences of going home with their infant following first stage cardiac surgery for single ventricle heart condition	10.1136/archdischild-2015-308599.20	Main search	01. Not AUS/NZ;
Gaskin 2015	Parents experiences of going home with their infant following first stage cardiac surgery for single ventricle heart condition	10.1136/archdischild-2015-308599.20	Main search	01. Not AUS/NZ;

Gaskin 2015	Transition from hospital to home: Psychosocial adaptation and adjustment in parents of infants with single ventricle heart conditions	10.1136/archdischild-2015-308599.11	Main search	01. Not AUS/NZ;
Gassner 2003	Aerobic exercise and the post myocardial infarction patient: A review of the literature	10.1016/S0147-9563%2803%2900039-6	Main search	03. Not SH;
Gill 2016	Feeling angry about current health status: using a population survey to determine the association with demographic, health and social factors	10.1186/s12889-016-3232-5	Main search	02. Not CVD;
Gliksman 1995	Social support, marital status and living arrangement correlates of cardiovascular disease risk factors in the elderly	10.1016/0277-9536%2894%2900149-N	Main search	02. Not CVD;

Glozier 2013	Psychosocial risk factors for coronary heart disease		Main search	04. CVD & SH mentioned but no outcome;
Glozier 2014	The national heart foundation of Australia consensus statement on psychosocial risk factors for coronary heart disease	10.1016/j.gheart.2014.03.1930	Main search	04. CVD & SH mentioned but no outcome;
Graven 2011	From rehabilitation to recovery: Protocol for a randomised controlled trial evaluating a goal-based intervention to reduce depression and facilitate participation post-stroke	10.1186/1471-2377-11-73	Main search	05. Intervention;

Graves 2009	Cost-Effectiveness of an Intervention to Reduce Emergency Re-Admissions to Hospital among Older Patients	10.1371/journal.pone.0007455	Main search	05. Intervention;
Grey 2010	A comparative analysis of cardiovascular disease risk profiles of five Pacific ethnic groups assessed in New Zealand primary care practice: PREDICT CVD-13		Main search	03. Not SH;
Grohn 2012	The first 3-months post-stroke: What facilitates successfully living with aphasia?	10.3109/17549507.2012.692813	Main search	02. Not CVD;
Gu 2016	Identifying ehealth opportunities to support medication adherence - Findings of a focus group study	10.3233/978-1-61499-645-3-150	Main search	04. CVD & SH mentioned but no outcome;

Hall 2015	Improving longer term outcomes post stroke: Exploring the barriers and facilitators that influence unmet need, life quality and participation after stroke	10.1111/ijs.12585	Main search	01. Not AUS/NZ;
Hammash 2019	Perceived control and quality of life among recipients of implantable cardioverter defibrillator	10.1016/j.aucc.2018.08.005	Main search	07. Outcome not hospital utilisation
Hammond 2008	Factors associated with persistent risk of depression in older people following discharge from an acute cardiac unit	10.1017/S1041610208007138	Main search	07. Outcome not hospital utilisation
Hancock 2017	Rational clinical evaluation of suspected acute coronary syndromes: The value of more information	10.1111/1742-6723.12819	Main search	03. Not SH;

Hand 1996	Older adults with lifelong intellectual handicap in New Zealand: prevalence, disabilities and implications for regional health authorities		Main search	02. Not CVD;
Harris 2010	How do we manage patients who become unemployed?		Main search	02. Not CVD;
Hawkes 2009	Randomised controlled trial of a secondary prevention program for myocardial infarction patients ('ProActive Heart'): Study protocol. Secondary prevention program for myocardial infarction patients	10.1186/1471-2261-9-16	Main search	05. Intervention;
Hawkes 2013	Predictors of physical and mental health-related quality of life outcomes among myocardial infarction patients	10.1186/1471-2261-13-69	Main search	05. Intervention;
Hawthorne 2008	Perceived social isolation in a community sample: its prevalence and correlates with aspects of peoples' lives	10.1007/s00127-007-0279-8	Main search	02. Not CVD;

Haynes 2019	Community-based participatory action research on rheumatic heart disease in an Australian Aboriginal homeland: Evaluation of the 'On track watch' project	10.1016/j.evalprogplan.2019.02.010	Main search	03. Not SH;
Hein 2013	Myocardial infarction in singapore: Ethnic variation in evidence-based therapy and its association with socioeconomic status, social network size and perceived stress level	10.1016/j.hlc.2013.04.119	Main search	01. Not AUS/NZ;
Hepburn-Brown 2019	Early decision-making in acute pulmonary embolism: a retrospective clinical audit	10.1111/imj.14042	Main search	03. Not SH;
Hodge 2013	Social connectedness and predictors of successful ageing	10.1016/j.maturitas.2013.05.002	Main search	02. Not CVD;

Howe 2012	'You needed to rehab ... families as well': family members' own goals for aphasia rehabilitation		Main search	02. Not CVD;
Hsu-Hage 2001	A qualitative investigation into the use of health services among Melbourne Chinese		Main search	02. Not CVD;
Hua 2017	Validation and recalibration of the Framingham cardiovascular disease risk models in an Australian Indigenous cohort	10.1177/2047487317722913	Main search	03. Not SH;
Huffman 2010	Cardiovascular Health in Indigenous Communities: Successful Programs	10.1016/j.hlc.2010.02.013	Main search	04. CVD & SH mentioned but no outcome;

Hutchinson 2015	Relationship between health-related quality of life, comorbidities and acute health care utilisation, in adults with chronic conditions	10.1186/s12955-015-0260-2	Main search	04. CVD & SH mentioned but no outcome;
Hyun 2017	Gender inequalities in cardiovascular risk factor assessment and management in primary healthcare	10.1136/heartjnl-2016-310216	Main search	03. Not SH;
Ingles 2007	Sudden cardiac death in the young: a clinical genetic approach		Main search	03. Not SH;
Ingles 2014	Medication non-compliance in patients with hypertrophic cardiomyopathy	10.1177/1474515114521363	Main search	07. Outcome not hospital utilisation

Iyngkaran 2016	Self Managing Heart Failure in Remote Australia - Translating Concepts into Clinical Practice	10.2174/1573403x12666160703183001	Main search	06.Review;
Jackson 2017	Psychosocial Screening and Assessment Practice within Cardiac Rehabilitation: A Survey of Cardiac Rehabilitation Coordinators in Australia	10.1016/j.hlc.2016.04.018	Main search	04. CVD & SH mentioned but no outcome;
Jacobs 2011	Does being elderly and living alone impact on outcomes following participation in a cardiac rehabilitation program?	10.1016/j.hlc.2011.05.606	Main search	04. CVD & SH mentioned but no outcome;

Jeacocke 2002	Adopting guideline review criteria as part of a regional project to improve heart failure management in general practice	10.1108/14664100210427615	Main search	03. Not SH;
Jeon 2010	Achieving a balanced life in the face of chronic illness		Main search	02. Not CVD;
Jeremy 2010	Improving Cardiovascular Care for Indigenous Populations	10.1016/j.hlc.2010.02.015	Main search	06.Review;
Kahl 2016	Quality of life in adults with congenital heart disease: What matters?	10.21037/jtd.2016.10.66	Main search	03. Not SH;
Karageorge 2020	Previous experience and walking capacity predict community outings after stroke: An observational study	10.1080/09593985.2018.1484829	Main search	04. CVD & SH mentioned

				but no outcome;
Karata ÅŸ 2017	Perceived social support and psychosocial adjustment in patients with coronary heart disease	10.1111/ijn.12558	Main search	01. Not AUS/NZ;
Kendall 2007	Recovery following stroke: The role of self-management education	10.1016/j.socscimed.2006.09.012	Main search	05. Intervention;
Keneally 2012	A 'whole of system' approach to compare options for CVD interventions in Counties Manukau	10.1111/j.1753-6405.2011.00812.x	Main search	03. Not SH;
Kerr 2019	A unified national cardiovascular disease (CVD) risk generator is required to address equity in the management of CVD risk in clinical practice in New Zealand		Main search	03. Not SH;
Killey 2014	Paths to work after stroke in Australia	10.1017/BrImp.2014.18	Main search	03. Not SH;

Kiropoulos 2012	Increased psychosocial stress in Greek-born immigrants compared to Anglo-Australians with coronary heart disease: the healthy heart, healthy mind study	10.1016/j.hlc.2012.07.018	Main search	04. CVD & SH mentioned but no outcome;
Knight 2017	Developing a synthetic national population to investigate the impact of different cardiovascular disease risk management strategies: A derivation and validation study	10.1371/journal.pone.0173170	Main search	03. Not SH;
Kowal 2010	Enduring dilemmas of Indigenous health. "You're always hearing about the stats ... death happens so often": new perspectives on barriers to Aboriginal participation in cardiac rehabilitation. Comment		Main search	04. CVD & SH mentioned but no outcome;

Kritharides 2010	Overview and Determinants of Cardiovascular Disease in Indigenous Populations	10.1016/j.hlc.2010.02.017	Main search	04. CVD & SH mentioned but no outcome;
Lanyon 2018	Exploring participant perspectives of community aphasia group participation: from "I know where I belong now" to "Some people didn't really fit in"	10.1080/02687038.2017.1396574	Main search	02. Not CVD;
Lauckner 2016	Peer support for people with chronic conditions in rural areas: a scoping review		Main search	02. Not CVD;
Lauder 2006	Social capital, age and religiosity in people who are lonely		Main search	02. Not CVD;

Lauder 2006	A comparison of health behaviours in lonely and non-lonely populations	10.1080/13548500500266607	Main search	02. Not CVD;
Lawrence 2017	Yoga for stroke rehabilitation	10.1002/14651858.CD011483.pub2	Main search	05. Intervention;
Leigh 2004	The clinical support systems program		Main search	02. Not CVD;
Leung 2010	Geographic issues in cardiac rehabilitation utilization: A narrative review	10.1016/j.healthplace.2010.08.004	Main search	01. Not AUS/NZ;
Li 2016	Impact of socioeconomic and risk factors on cardiovascular disease and type II diabetes in Australia: comparison of results from longitudinal and cross-sectional designs	10.1136/bmjopen-2015-010215	Main search	03. Not SH;

Lieberman	LIEBERMAN , LANI, B.A., MARTA MEANA, Ph.D., and DONNA STEWART, M.D., F.R.C.P.(C)		References	01. Not AUS/NZ;
Liu 2016	Risk factors for obstructive sleep apnea are prevalent in people with psychosis and correlate with impaired social functioning and poor physical health	10.3389/fpsyt.2016.00139	Main search	04. CVD & SH mentioned but no outcome;
Lo 2012	Pediatric stroke outcome measure predicts cognitive and functional deficits after childhood ischemic stroke		Main search	04. CVD & SH mentioned but no outcome;

Lobstein 2004	Obesity in children and young people: A crisis in public health		Main search	02. Not CVD;
Longman 2012	Frequent hospital admission of older people with chronic disease: A cross-sectional survey with telephone follow-up and data linkage	10.1186/1472-6963-12-373	Main search	04. CVD & SH mentioned but no outcome;
Lord 2008	How feasible is the attainment of community ambulation after stroke? A pilot randomized controlled trial to evaluate community-based physiotherapy in subacute stroke	10.1177/0269215507081922	Main search	05. Intervention;
Lovell 2010	Telehealth technologies for managing chronic disease - Experiences from Australia and the UK	10.1109/IEMBS.2010.5626312	Main search	03. Not SH;

Lynch 2016	Education-only versus a multifaceted intervention for improving assessment of rehabilitation needs after stroke; a cluster randomised trial	10.1186/s13012-016-0487-2	Main search	03. Not SH;
Lynch 2018	Activity monitors for increasing physical activity in adult stroke survivors	10.1002/14651858.CD012543.pub2	Main search	05. Intervention;
Macniven 2012	Barriers and enablers to physical activity among older Australians who think they are insufficiently active	10.1016/j.jsams.2012.11.110	Main search	02. Not CVD;
Madsen 2013	'This is a forever project': supporting lifestyle changes in a regional Queensland community-based cardiac rehabilitation program	10.1071/PY11137	Main search	04. CVD & SH mentioned but no outcome;

Maiorana 2015	Reducing inequities in aboriginal Australian heart health through culturally specific cardiac rehabilitation	10.1177/2047487315586733	Main search	03. Not SH;
Maneze 2018	Negotiating health and chronic illness in Filipino-Australians: a qualitative study with implications for health promotion	10.1080/13557858.2017.1294656	Main search	02. Not CVD;
Marmot 2000	Social determinants of health: From observation to policy		Main search	02. Not CVD;
Marsden 2010	A multidisciplinary group programme in rural settings for community-dwelling chronic stroke survivors and their carers: a pilot randomized controlled trial	10.1177/0269215509344268	Main search	05. Intervention;
Martin 2012			References	01. Not AUS/NZ;

McBride 2016	McBride SA Aboriginal Cardiovascular Health Profile 2016		Author contact	03. Not SH;
McCluskey 2015	Compliance with Australian stroke guideline recommendations for outdoor mobility and transport training by post-inpatient rehabilitation services: An observational cohort study	10.1186/s12913-015-0952-7	Main search	03. Not SH;
McKenna 2009	Comparison of time use, role participation and life satisfaction of older people after stroke with a sample without stroke	10.1111/j.1440-1630.2007.00728.x	Main search	03. Not SH;
McLachlan 2010	Equity of access to CVD risk management using electronic clinical decision support in the coronary care unit	10.1016/j.ejcnurse.2010.01.007	Main search	03. Not SH;

McLaughlin 2000	Travelling sales: An occupational hazard?		Main search	02. Not CVD;
Meyer 2013	Differentiating between trust and dependence of patients with coronary heart disease: furthering the sociology of trust	10.1080/13698575.2013.776017	Main search	03. Not SH;
Milligan 1997	Health-related behaviours and psycho-social characteristics of 18 year-old Australians		Main search	02. Not CVD;
Mitchell 1992	A cross-cultural assessment of perceived health problems in the elderly		Main search	04. CVD & SH mentioned but no outcome;

Mitchell 2014	The healthy neighbourhood audit instrument: Understanding the environmental and socio-cultural conditions to support healthy, happy and resilient residential communities		Main search	02. Not CVD;
Moorley 2015	Life after stroke: Releasing the cultural hostage	10.1111/ijs.12584	Main search	01. Not AUS/NZ;
Morris 1991	The relationship between the perception of social support and post-stroke depression in hospitalized patients		Main search	07. Outcome not hospital utilisation
Murphy (2008			References	07. Outcome not hospital utilisation

Murphy 2013	Are poor health behaviours in anxious and depressed cardiac patients explained by sociodemographic factors?	10.1177/2047487312449593	Main search	07. Outcome not hospital utilisation
Murphy 2014	Red flags for persistent or worsening anxiety and depression after an acute cardiac event: a 6-month longitudinal study in regional and rural Australia	10.1177/2047487313493058	Main search	07. Outcome not hospital utilisation
Myburgh 2018	Coping and Cardiac Troponin T â€" A Risk for Hypertension and Sub-Clinical ECG Left Ventricular Hypertrophy: The SABPA Study	10.1016/j.hlc.2018.05.101	Main search	02. Not CVD;
Nelson 2019	Developing cardiovascular risk prediction models for Australia	10.5694/mja2.50010	Main search	03. Not SH;
O'Mara 2012	The spirit of the tent embassy: 40 years on indigenous self-determination is essential to health and wellbeing	10.5694/mja12.10829	Main search	02. Not CVD;

Paisley 2008	Dietary change: What are the responses and roles of significant others?	10.1016/j.jneb.2007.04.374	Main search	02. Not CVD;
Patel 2014	Understanding the impact of a multifaceted quality improvement intervention to improve cardiovascular disease risk management in Australian primary health care: The TORPEDO study process evaluation	10.1016/j.gheart.2014.03.2002	Main search	05. Intervention;
Patel 2014	A multifaceted quality improvement intervention for CVD risk management in Australian primary healthcare: a protocol for a process evaluation	10.1186/s13012-014-0187-8	Main search	05. Intervention;
Patel 2017	Impact of sustained use of a multifaceted computerized quality improvement intervention for cardiovascular disease management in Australian primary health care	10.1161/JAHA.117.007093	Main search	05. Intervention;

Patel 2018	What drives adoption of a computerised, multifaceted quality improvement intervention for cardiovascular disease management in primary healthcare settings? A mixed methods analysis using normalisation process theory	10.1186/s13012-018-0830-x	Main search	03. Not SH;
Patterson 2009	Long-term stroke survivorsâ€™ needs and perceptions of an exercise maintenance model of care	10.12968/ijtr.2009.16.12.45422	Main search	07. Outcome not hospital utilisation
Patterson 2010	Stroke maintenance exercise group: Pilot study on daily functioning in long-term stroke survivors	10.1071/PY09055 10.1046/j.1365-2648.2000.01517.x	Main search	05. Intervention;
Paul 2005			References	03. Not SH;

Peiris 2009	An electronic clinical decision support tool to assist primary care providers in cardiovascular disease risk management: development and mixed methods evaluation		Main search	03. Not SH;
Peiris 2014	Effect of a multi-faceted quality improvement intervention to improve cardiovascular disease risk identification and management in australian primary health care: The torpedo cluster-randomised trial	10.1016/j.gheart.2014.03.1317	Main search	05. Intervention;
Peiris 2015	Effect of a computer-guided, quality improvement program for cardiovascular disease risk management in primary health care: The treatment of cardiovascular risk using electronic decision support cluster-randomized trial	10.1161/CIRCOUTCOMES.114.001235	Main search	05. Intervention;
Penn 2017	Intercultural aphasia: new models of understanding for Indigenous populations	10.1080/02687038.2016.1213788	Main search	02. Not CVD;

Petrie 1996	Role of patients' view of their illness in predicting return to work and functioning after myocardial infarction: longitudinal study		Main search	04. CVD & SH mentioned but no outcome;
Pettman 2008	Self-management for obesity and cardio-metabolic fitness: Description and evaluation of the lifestyle modification program of a randomised controlled trial	10.1186/1479-5868-5-53	Main search	05. Intervention;
Pier 2008	Identifying the health and mental health information needs of people with coronary heart disease, with and without depression		Main search	07. Outcome not hospital utilisation
Pit 2010	Health problems and retirement due to ill-health among Australian retirees aged 45-64 years	10.1016/j.healthpol.2009.09.003	Main search	02. Not CVD;

Pitama 2011	A Kaupapa Maori approach to a community cohort study of heart disease in New Zealand	10.1111/j.1753-6405.2011.00702.x	Main search	03. Not SH;
Provance 2019	Assessing Patient Preferences for Shared Decision-Making in Peripheral Artery Disease: Insights from the PORTRAIT Registry	10.1161/CIRCOUTCOMES.119.005730	Main search	01. Not AUS/NZ;
Quigley 2019	Are we there yet? Exploring the journey to quality stroke care for Aboriginal and Torres Strait Islander peoples in rural and remote Queensland	10.22605/RRH4850	Main search	04. CVD & SH mentioned but no outcome;
Quirk 2018	Predictors of Physical Activity Among Rural Adults Following Cardiac Rehabilitation	10.1037/rep0000232	Main search	07. Outcome not hospital utilisation

Raban al 2018	Performance of a Framingham cardiovascular risk model among Indians and Europeans in New Zealand and the role of body mass index and social deprivation	10.1136/openhrt-2018-000821	Main search	03. Not SH;
Ramsamy 2017	A retrospective audit of post discharge outcome for patients supported by 'acute coronary syndrome support network' to remote communities in the northern territory of australia	10.1016/j.hlc.2017.06.664	Main search	03. Not SH;
Ranta 2013	TIA management in rural and provincial New Zealand	10.1111/ijs.12141	Main search	04. CVD & SH mentioned but no outcome;
Ray 2001	Self-reported heart health behaviour patterns in a rural context		Main search	02. Not CVD;

Reilly 2008	Identifying psychosocial mediators of health amongst indigenous Australians for the Heart Health Project	10.1080/13557850801903046	Main search	04. CVD & SH mentioned but no outcome;
Riddell 2012	Cluster randomized controlled trial of a peer support program for people with diabetes: Study protocol for the Australasian peers for progress study	10.1186/1471-2458-12-843	Main search	02. Not CVD;
Riddell 2016	Cardiovascular risk outcome and program evaluation of a cluster randomised controlled trial of a community-based, lay peer led program for people with diabetes	10.1186/s12889-016-3538-3	Main search	02. Not CVD;

Robinson 1999			References	01. Not AUS/NZ;
Rosbergen 2017	Embedding an enriched environment in an acute stroke unit increases activity in people with stroke: a controlled before-after pilot study	10.1177/0269215517705181	Main search	05. Intervention;
Rosbergen 2019	The impact of environmental enrichment in an acute stroke unit on how and when patients undertake activities	10.1177/0269215518820087	Main search	05. Intervention;
Rosengren 2004			References	03. Not SH;

Rosland 2010			References	01. Not AUS/NZ;
Ryan 2017	A cross-sectional study of work-related and lifestyle factors associated with the health of Australian long distance commute and residential miners	10.1080/21679169.2017.1381324	Main search	02. Not CVD;
Sahle 2020	Association of Psychosocial Factors With Risk of Chronic Diseases: A Nationwide Longitudinal Study	10.1016/j.amepre.2019.09.007	Main search	04. CVD & SH mentioned but no outcome;
Sapuppo 2018	The unmet needs of young stroke survivors	10.1177/1747493018778666	Main search	07. Outcome not hospital utilisation

Scott 2004	Achieving better in-hospital and after-hospital care of patients with acute cardiac disease		Main search	05. Intervention;
Scott 2015	Body mass, cardiovascular risk and metabolic characteristics of young persons presenting for mental healthcare in Sydney, Australia	10.1136/bmjopen-2014-007066	Main search	02. Not CVD;
Shanks 2007	Author confirms		References	01. Not AUS/NZ;
Simons 1991	DUBBO STUDY OF THE ELDERLY - SOCIOLOGICAL AND CARDIOVASCULAR RISK-FACTORS AT ENTRY	10.1111/j.1445-5994.1991.tb01373.x	Main search	04. CVD & SH mentioned but no outcome;

Simons 2013	Impact of loneliness and living alone		Main search	04. CVD & SH mentioned but no outcome;
Sinnett 1978	Lifestyle, health and disease: a comparison between Papua New Guinea and Australia		Main search	02. Not CVD;
Ski 2007	Stroke: the increasing complexity of carer needs		Main search	02. Not CVD
Son 2016	Biopsychosocial predictors of coping strategies of patients postmyocardial infarction	10.1111/ijn.12465	Main search	07. Outcome not hospital utilisation

Son 2019	How do patients develop self-care behaviors to live well with heart failure?: A focus group interview study	10.1016/j.colegn.2018.12.004	Main search	01. Not AUS/NZ;
Spaderna 2017	Role of Depression and Social Isolation at Time of Waitlisting for Survival 8 Years After Heart Transplantation	10.1161/JAHA.117.007016	Main search	02. Not CVD
Spaeth 2018	Economic evaluation of point-of-care testing in the remote primary health care setting of Australia's Northern Territory	10.2147/CEOR.S160291	Main search	03. Not SH;
Speechly 2010	Patient and general practitioner attitudes to healthy lifestyle behaviours and medication following coronary heart disease: An exploratory study	10.1071/PY09011	Main search	07. Outcome not hospital utilisation
Stapelberg 2011	A topographical map of the causal network of mechanisms underlying the relationship between major depressive disorder and coronary heart disease	10.3109/00048674.2011.570427	Main search	04. CVD & SH mentioned

				but no outcome;
Strodl 2008	The 5-item mental health index predicts the initial diagnosis of nonfatal stroke in older women	10.1089/jwh.2007.0516	Main search	04. CVD & SH mentioned but no outcome;
Strodl 2013	A history of heart interventions moderates the relationship between psychological variables and the presence of chest pain in older women with self-reported coronary heart disease	10.1111/bjhp.12011	Main search	07. Outcome not hospital utilisation
Stuart 2014	A telephone-supported cardiovascular lifestyle programme (CLIP) for lipid reduction and weight loss in general practice patients: a randomised controlled pilot trial	10.1017/S1368980013000220	Main search	05. Intervention;

Sturm 2004			References	04. CVD & SH mentioned but no outcome;
Tamplin 2013	'Stroke a chord': The effect of singing in a community choir on mood and social engagement for people living with aphasia following a stroke	10.3233/NRE-130916	Main search	02. Not CVD;
Tavener 2015	Acknowledging How Older Australian Women Experience Life After Stroke: How Does the WHO 18-Item Brief ICF Core Set for Stroke Compare?	10.1080/07399332.2015.1055747	Main search	07. Outcome not hospital utilisation
Taylor 2014	Implementing the evidence: From presumption to training	10.1111/ijs.12334	Main search	04. CVD & SH

				mentioned but no outcome;
Thompson 2017	Gender disparities in cardiovascular disease prevention	10.1136/heartjnl-2016-310788	Main search	03. Not SH;
Thurston 2008	What Happens Next? The Role of Cardiac Rehabilitation in Total Patient Care	10.1016/j.hlc.2008.09.005	Main search	05. Intervention;
Tibby 2010	Establishment of an Innovative Specialist Cardiac Indigenous Outreach Service in Rural and Remote Queensland	10.1016/j.hlc.2010.02.023	Main search	03. Not SH;

Tidem an 2014	Impact of a regionalised clinical cardiac support network on mortality among rural patients with myocardial infarction		Main search	03. Not SH;
Tse 2017	Reduction in retained activity participation is associated with depressive symptoms 3 months after mild stroke: An observational cohort study	10.2340/16501977-2184	Main search	07. Outcome not hospital utilisation
Tse 2018	Longitudinal changes in activity participation in the first year post-stroke and association with depressive symptoms	10.1080/09638288.2018.1471742	Main search	07. Outcome not hospital utilisation
Tsuchihashi-Makaya 2009			References	01. Not AUS/NZ;
Tully 2014	Routine depression screening after cardiac surgery simply misses those that need it most: Impact of missed cases on	10.1177/1474515114521363	Main search	04. CVD & SH

	hospital resource utilization, cardiac outcomes, depression and quality of life		mentioned but no outcome;
Unsworth 1997a	Agreement Between Occupational Therapists and Clients with Stroke on Three	Author contact	03. Not SH;
Unsworth 1997b	Decision polorization	Author contact	04. CVD & SH mentioned but no outcome;

Unsworth 2002	Examining the Evidence Base for Occupational Therapy with Clients following Stroke		Author contact	03. Not SH;
Unsworth 2005	A Comparison of Client Outcomes from Two Acute Care Neurological Services using Self-care Data from the Australian Therapy Outcome Measures for Occupational Therapy (AusTOMs-OT)		Author contact	03. Not SH;
Unsworth 2019	Preliminary Screening Recommendations for Patients at Risk of Depression and/or Anxiety more than 1 year Poststroke	10.1016/j.jstrokecerebrovasdis.2019.03.014	Main search	07. Outcome not hospital utilisation
Vallesi 2018	"In Their Own Voice"-Incorporating Underlying Social Determinants into Aboriginal Health Promotion Programs	10.3390/ijerph15071514	Main search	02. Not CVD;
Vogt 1992			References	01. Not AUS/NZ;

Volz 2011			References	01. Not AUS/NZ;
VonDohren 2013	Taking therapeutic leisure and recreation seriously during stroke recovery	10.1111/ijs.12172	Main search	04. CVD & SH mentioned but no outcome;
Wang 2010	The prevalence and predictors of anxiety and depression in adolescents with heart disease	10.1111/j.1445-5994.2010.02186.x	Main search	07. Outcome not hospital utilisation
Wang 2011	Psychosocial functioning in adolescents with heart disease	10.1016/j.hlc.2011.05.599	Main search	07. Outcome not hospital utilisation

Ward 2011	With good intentions: complexity in unsolicited informal support for Aboriginal and Torres Strait Islander peoples. A qualitative study	10.1186/1471-2458-11-686	Main search	02. Not CVD;
Wells 2011	The predictive power of family history of premature CVD in a large new zealand primary care cohort	10.1093/eurheartj/ehr323	Main search	03. Not SH;
Wells 2017	Cohort Profile: The PREDICT cardiovascular disease cohort in New Zealand primary care (PREDICT-CVD 19)	10.1093/ije/dyv312	Main search	03. Not SH;
Westbrook 1993	Attitudes towards disabilities in a multicultural society		Main search	02. Not CVD
Westlake 2002			References	01. Not AUS/NZ;

White 2007	Community-dwelling stroke survivors: function is not the whole story with quality of life		Main search	04. CVD & SH mentioned but no outcome;
White 2008	The occupational experience of stroke survivors in a community setting	10.3928/15394492-20080901-05	Main search	04. CVD & SH mentioned but no outcome;
White 2008			References	04. CVD & SH mentioned

				but no outcome;
White 2009	Exploring poststroke mood changes in community-dwelling stroke survivors: A prospective, longitudinal, mixed methods study	10.1111/j.1747-4949.2009.00306.x	Main search	07. Outcome not hospital utilisation
White 2010	Exploring post-stroke mood changes in community-dwelling stroke survivors: A longitudinal cohort study	10.1111/j.1747-4949.2010.00458-3.x	Main search	07. Outcome not hospital utilisation
White 2016	Predictors of health-related quality of life in community-dwelling stroke survivors: a cohort study	10.1093/fampra/cmw011	Main search	07. Outcome not hospital utilisation

Wilson 1993	The Good Heart, Good Life survey: self-reported cardiovascular disease risk factors, health knowledge and attitudes among Greek Australians in Sydney	10.1111/j.1753-6405.1993.tb00138.x	Main search	02. Not CVD;
Worrall-Carter 2005	The experiences and adjustments of women following their first acute myocardial infarction		Main search	04. CVD & SH mentioned but no outcome;
Wu 2013			References	01. Not AUS/NZ;
Yarmo-	The heart of the matter: Health status of aged care clients receiving home- and community-based care	10.4061/2010/275303	Main search	02. Not CVD;

Roberts 2010				
Yusuf 2004			References	03. Not SH;
Zecchin 2016	Cardiac rehabilitation for patients with spontaneous coronary artery dissection	10.1016/j.hlc.2016.06.768	Main search	04. CVD & SH mentioned but no outcome;
Zhang 2017	Using the Think Aloud™ Technique to Explore Quality of Life Issues During Standard Quality-of-Life Questionnaires in Patients With Atrial Fibrillation	10.1016/j.hlc.2016.05.121	Main search	05. Intervention;
